# Supplementary material for: Two distinct catalytic pathways for GH43 xylanolytic enzymes unveiled by X-ray and QM/MM simulations
Source: Nat Commun. 2021 Jan 14;12:367. doi: 10.1038/s41467-020-20620-3 (PMC7809346; doi:10.1038/s41467-020-20620-3)
Supplement: Supplementary file 1 — Supplementary Information [file 41467_2020_20620_MOESM1_ESM.pdf]

SUPPLEMENTARY INFORMATION

Two distinct catalytic pathways for GH43 xylanolytic enzymes unveiled by X-ray and QM/MM simulations

Mariana A. B. Morais[1,2], Joan Coines[2], Mariane N. Domingues[1], Renan A. S. Pirolla[1], Celisa C. C. Tonoli[3], Camila R. Santos[1], Jessica B. L. Correa[1], Fabio C. Gozzo[4],

Carme Rovira[2,5,*], Mario T. Murakami[1,*]

[1] Brazilian Biorenewables National Laboratory (LNBR), Brazilian Center for Research in Energy and Materials (CNPEM), Campinas 13083-100, Brazil.

[2] Departament de Química Inorgànica i Orgànica & Institut de Química Teórica i Computacional (IQTCUB), Universitat de Barcelona, Barcelona 08028, Spain.

[3] Brazilian Biosciences National Laboratory (LNBio), Brazilian Center for Research in Energy and Materials (CNPEM), Campinas 13083-100, Brazil.

[4] Dalton Mass Spectrometry Laboratory, Institute of Chemistry, University of Campinas, Campinas 13083-970, Brazil.

[5] Institució Catalana de Recerca i Estudis Avançats (ICREA), Barcelona 08010, Spain.

[*] Correspondence and requests should be addressed to: M.T.M. (e-mail: mario.murakami@lnbr.cnpem.br) or to C.R. (e-mail: c.rovira@ub.edu).

Supplementary Figures

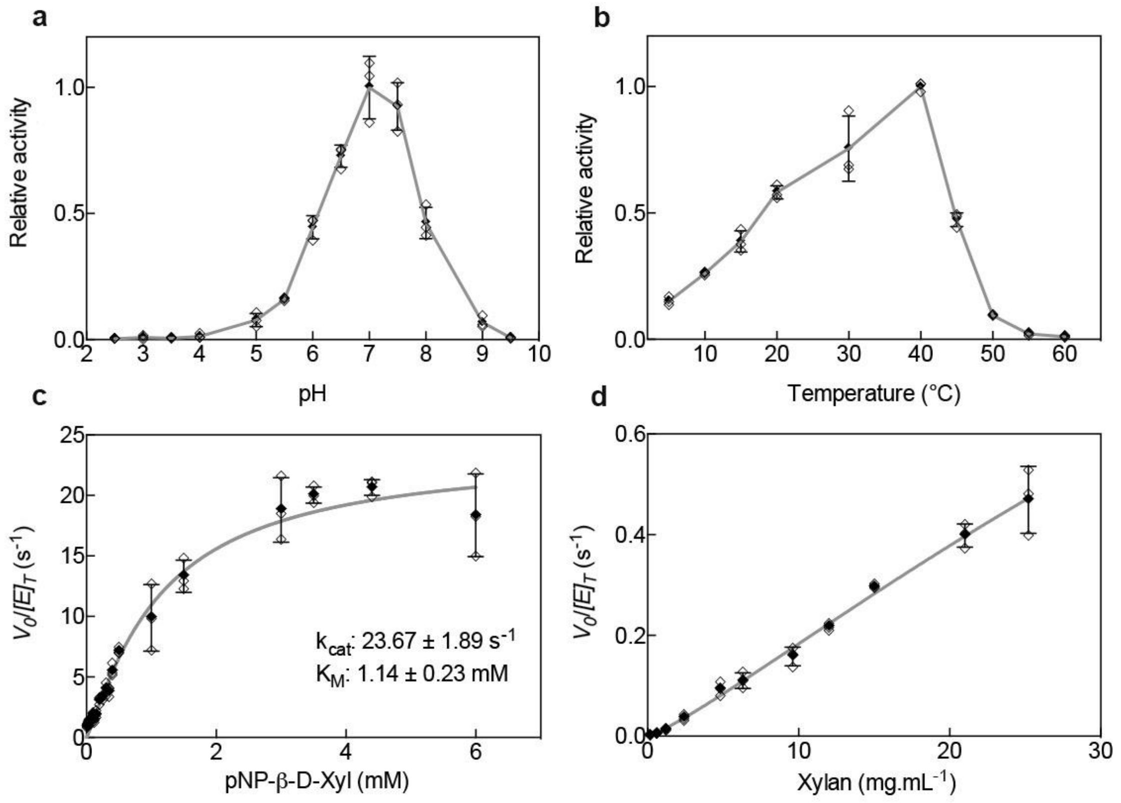

Supplementary Fig.1: Enzymatic characterization of XacGH43_1 by colorimetric methods. a-b Relative activity of XacGH43_1 over pNP-Xyl in function of pH (**a**) and temperature (**b**). XacGH43_1 displays maximum activity at pH 7.0 and 40 ^oC. **c** Kinetic curve and parameters of pNP-Xyl hydrolysis by XacGH43_1. **d** Activity of XacGH43_1 against different xylan concentrations. In (**d**), kinetic parameters were not calculated since the saturation was not reached. Results are expressed as mean ± SD from three independent experiments. Data points are shown as empty symbols. In (**c-d**), assays were performed at 40 ^oC, pH 7.0 with 6 mM of $CaCl_2$. Source data are provided as a source data file.

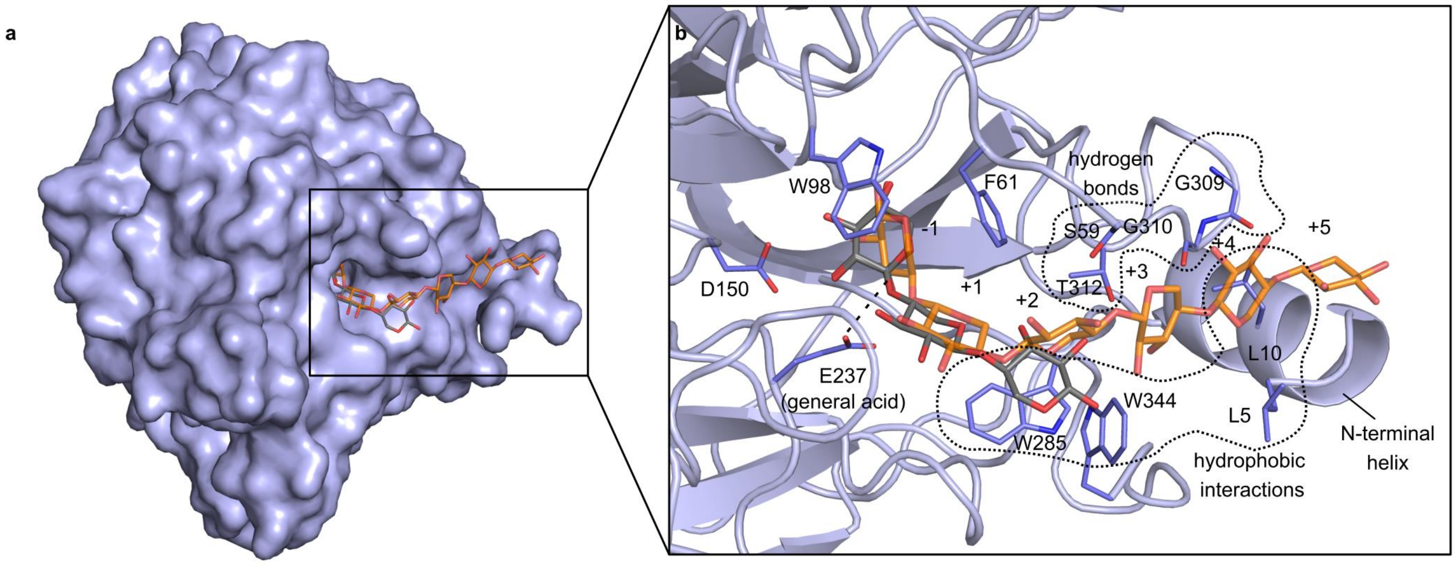

Supplementary Fig.2: Crystallographic XacGH43_1 + X3 complex and X6 docking. a Representation of XacGH43_1 surface, showing X3 (crystal) and X6 (docked using AutoDock Vina, as described in Methods) as sticks, with grey and orange carbon atoms, respectively. **b** XacGH43_1 as cartoon and sticks, highlighting residues involved in X3 or X6 stabilization by hydrophobic interactions or hydrogen bonds. The N-terminal helix, accounted to the extended topology of the active site, is indicated.

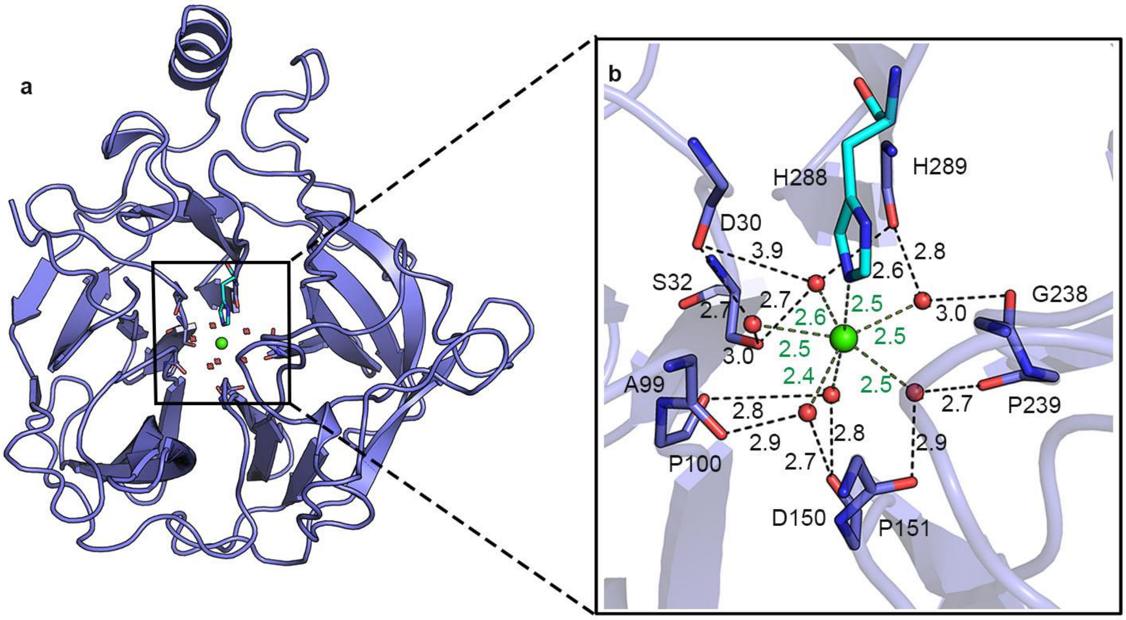

Supplementary Fig.3: Ca^{2+} coordination in XacGH43_1 crystal structure. a Cartoon representation of XacGH43_1 showing the Ca^{2+} (green sphere) and its coordinating waters (red spheres). **b** Ca^{2+} (green sphere) and its coordinating waters (red spheres) and residues (sticks). Main-chain carbonyl groups coordinating the water molecules are also represented (sticks). Distances are represented in angstroms

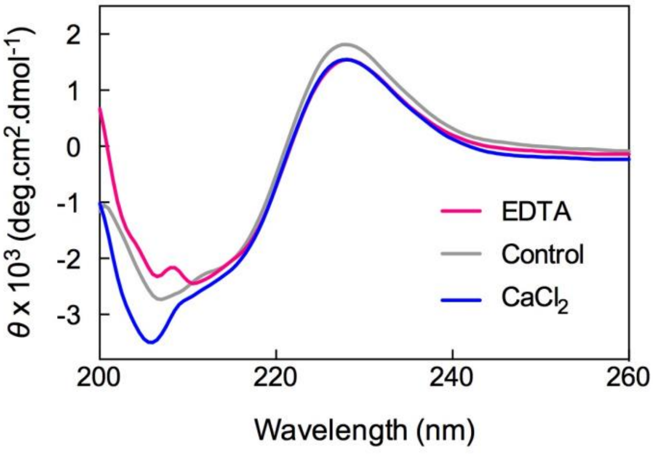

Supplementary Fig.4: Circular dichroism spectra of XacGH43_1 at 20 ºC. Samples were analyzed without additives (grey), with the addition of EDTA (pink) or $CaCl_2$ (blue). Source data are provided as a source data file.

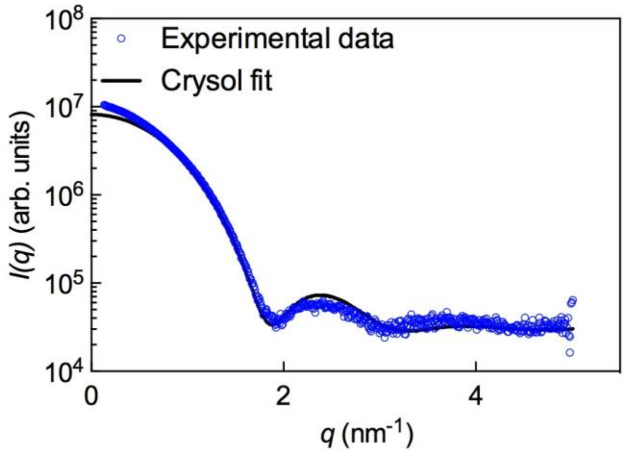

Supplementary Fig.5: XacGH43_1 SAXS data. Experimental scattering curve obtained by SAXS (blue circles) and theoretical scattering curve calculated from the XacGH43_1 crystallographic coordinates with CRYSOL (black line). Source data are provided as a source data file.

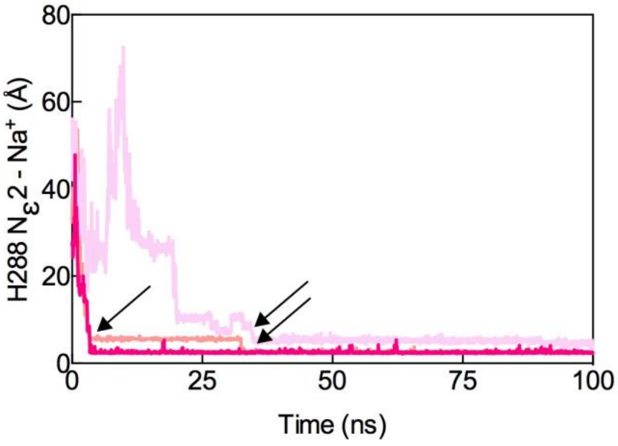

Supplementary Fig.6: Na^+ spontaneously entering into the metal-binding site during classical XacGH43_1 MD simulations without Ca^{2+}. Distance between the His288 $N_\varepsilon 2$ atom and the Na^+ that enters into the cation-binding site of XacGH43_1 during classical MD simulations where the Ca^{2+} had been initially replaced by a water molecule. The arrows indicate approximately when Na^+ enters into the metal-binding site. The different shades of pink correspond to three independent simulations. Source data are provided as a source data file.

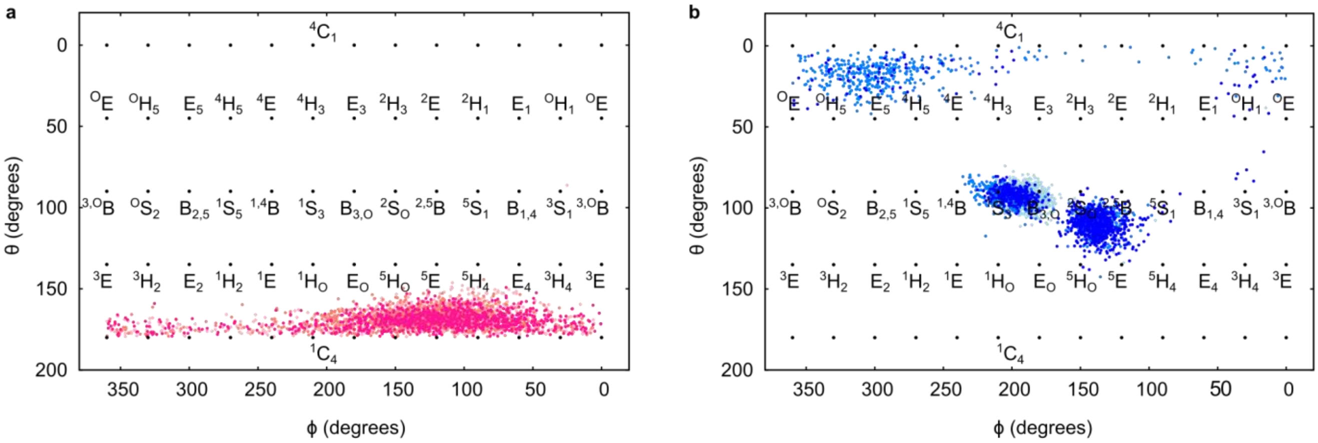

Supplementary Fig.7: Conformations of the -1 xylosyl residue during classical MD simulations. Mercator representation of the classical MD simulations of XacGH43_1 with water (replacing the Ca^{2+}) followed by the Na^{+} entrance (**a**) or with Ca^{2+} (**b**). The three independent simulations are shown in different shades of pink or blue for (**a**) and (**b**), respectively. Source data are provided as a source data file.

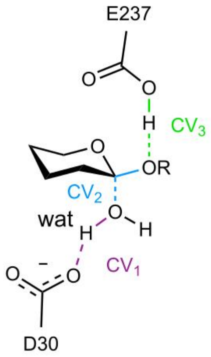

Supplementary Fig.8: Schematic representation of the collective variables used for *ab initio* metadynamics to monitor the reaction (hydrolysis). The first CV (CV_1 - purple) corresponds to the difference between H_{wat}–O_{wat} and O_{Asp30}–H_{wat} distances (wat = catalytic water). The second CV (CV_2 - blue) was taken as the difference between $C1_{xylosyl}$–$O4_{xylosyl}$ and O_{wat}–$C1_{xylosyl}$ distances, while the third CV (CV_3 - green) was taken as the difference between H_{Glu237}–O_{Glu237} and $O1_{xylosyl}$–H_{Glu237} distances.

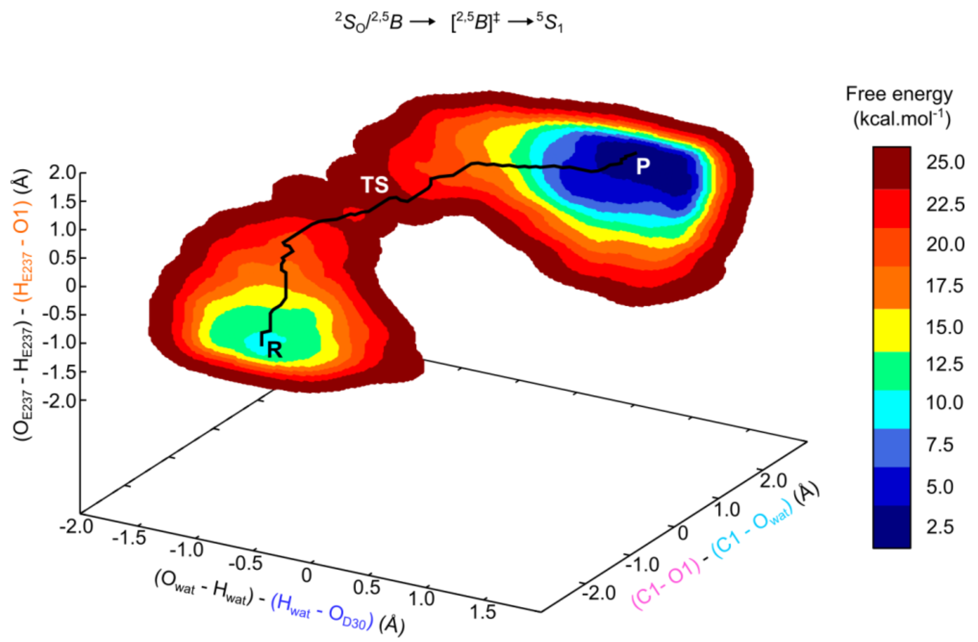

Supplementary Fig.9: FEL obtained for the $^2S_O/^{2,5}B \rightarrow [^{2,5}B]^‡ \rightarrow {}^5S_1$ catalytic itinerary by *ab initio* metadynamics. The minimum free energy pathway is indicated with a black line on the three-dimensional FEL. Reactant (R), transition state (TS) and product (P) states are indicated.

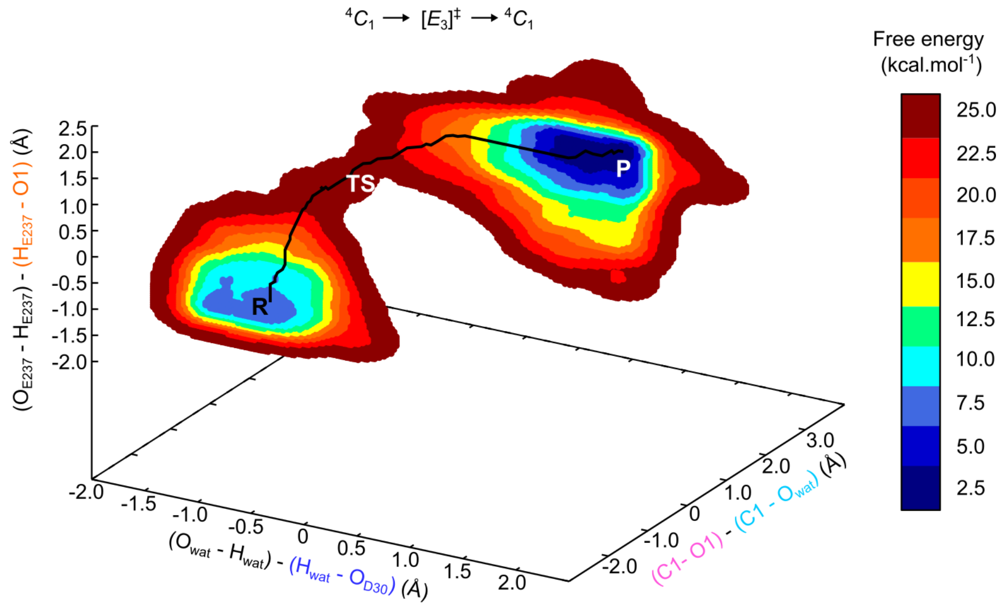

Supplementary Fig.10: FEL obtained for the $^4C_1 \rightarrow [E_3]^‡ \rightarrow {}^4C_1$ catalytic itinerary by *ab initio* metadynamics. The minimum free energy pathway is indicated with a black line on the three-dimensional FEL. Reactant (R), transition state (TS) and product (P) states are indicated.

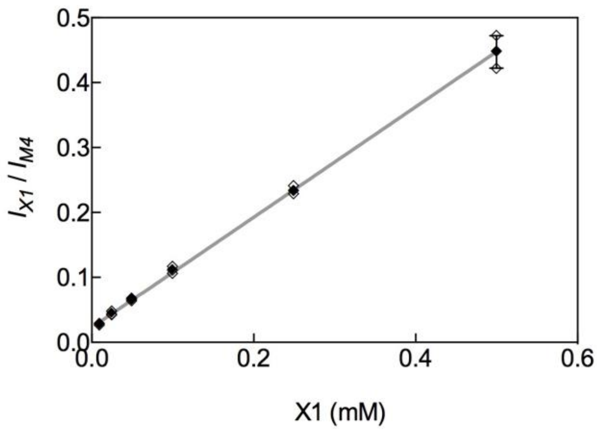

Supplementary Fig.11: Calibration curve to determine the concentrations of the products of enzymatic reactions by mass spectrometry. The intensity of the reaction product (X1) was divided by the internal standard intensity from mannotetraose (M4) (I_{X1}/I_{M4}). Results are expressed as mean ± SD from three independent experiments. Data points are shown as empty symbols. Source data are provided as a source data file.

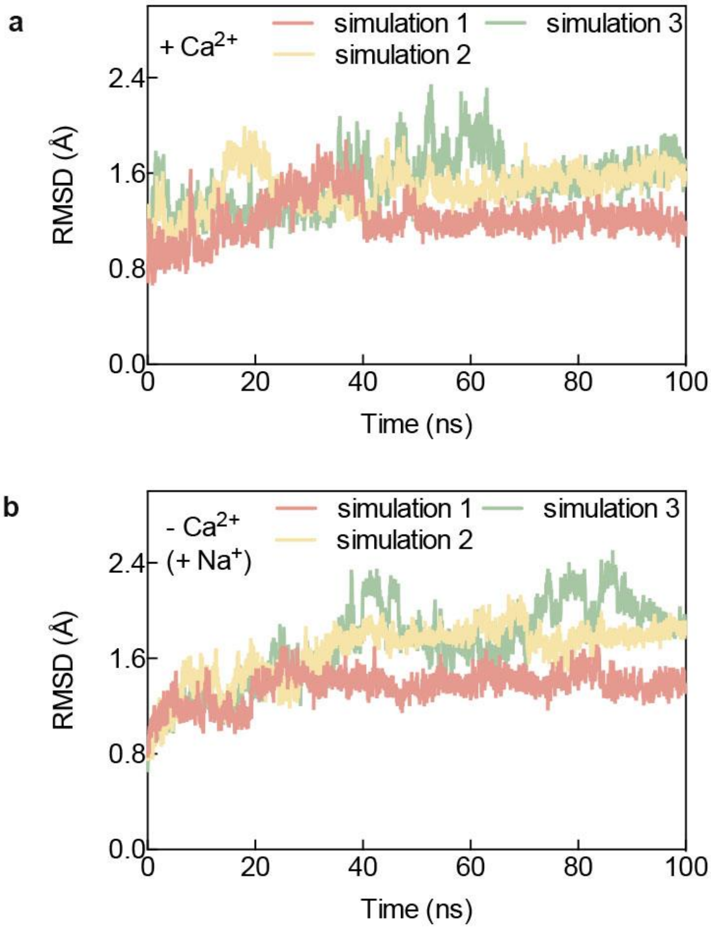

Supplementary Fig.12: Protein backbone RMSD fluctuations along classical MD trajectories of the xylotriose complex. a Simulations were performed in the presence of Ca^{2+} bound in the metal binding site. **b** Ca^{2+} was replaced by a water molecule, followed by its spontaneous substitution by a Na^{+} during the simulation. Three independent simulations with 100 ns of production were performed in **a,b**. Source data are provided as a source data file.

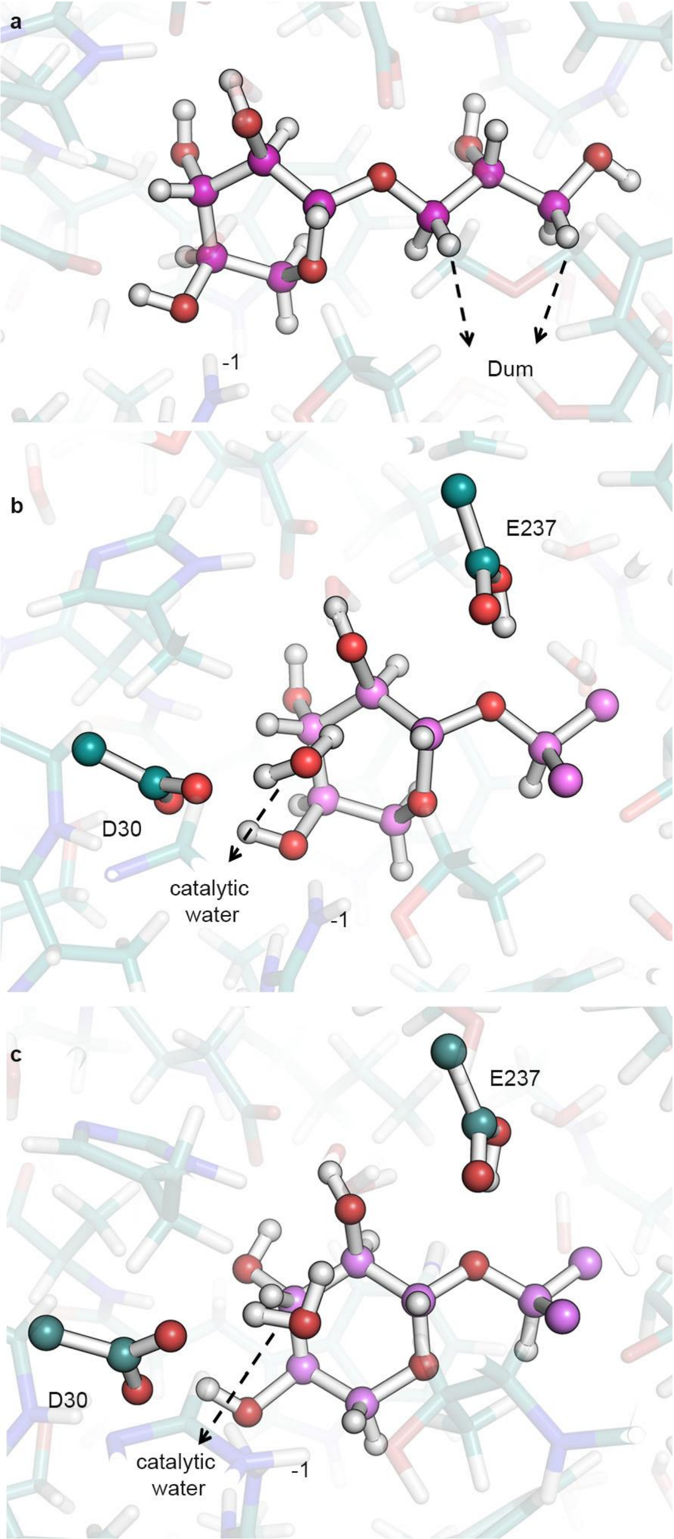

Supplementary Fig.13: QM regions of XacGH43_1 used in the QM/MM calculations. Coordinates used for QM/MM calculations and *ab initio* metadynamics to analyze the -1 xylosyl puckering coordinates (**a**), the reaction starting from the distorted $^{2}S_{O}/^{2,5}B$ conformation (**b**) and from the $^{4}C_{1}$ conformation (**c**). The dummy atoms introduced in (**a**) are indicated (Dum).

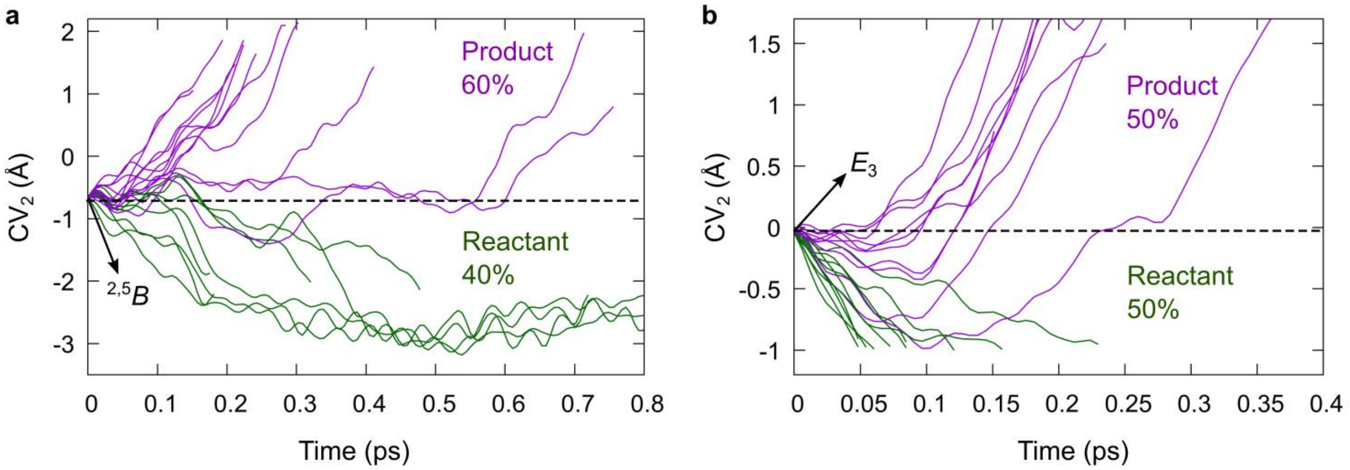

Supplementary Fig.14: Isocommittor analysis of transition states. a Trajectories projected on CV_2 (nucleophilic attack) starting from a representative frame of the TS $^{2,5}B$. From 20 independent simulations, 60% of the trajectories converged to the product and 40% to the reactant. **b** Trajectories projected on CV_2 (nucleophilic attack) starting from a representative frame of the TS E_3. From 20 independent simulations, 50% of the trajectories converged to the product and 50% to the reactant. The product and reactant states were assigned according to the distances observed during metadynamics simulations. Source data are provided as a source data file.

Supplementary Tables

Supplementary Table 1: List of substrates tested with XacGH43_1.

pNP-derived substrates	Polymeric substrates
pNP- α-D- galactopyranoside	Arabinan
pNP- α-D- glucopyranoside	Arabinogalactan
pNP- α-D- mannopyranoside	Arabinoxylan
pNP- α-D- xylopyranoside	Carboxymethyl Cellulose 4M
pNP- α-L-arabinofuranoside	Curdlan
pNP- α-L-arabinopyranoside	Debranched arabinan
pNP- α-L-fucopyranoside	Galactan
pNP- α-L-ramnopyranoside	Galactomannan
pNP- β-D-cellobioside	Laminarin
pNP- β-D-fucopyranoside	Pectic Galactan
pNP- β-D-galactopyranoside	Pectic from Citrus
pNP- β-D-glucopyranoside	Polygalacturonic Acid
pNP- β-D-mannopyranoside	Pullulan
pNP- β-D-xylopyranoside	Rhamnogalacturonan
	Xantham
	Xylan
	Xyloglucan
	Locust
	2-hidroxyetil cellulose
	β - Glucan
	Avicel
	Chitosan
	Lichenan
	Linear, 1,5 Arabinan
	Mannan (linear)
	Mannan
	Pachyman
	Rhamnogalacturonan
	Starch from potato

Supplementary Table 2: Kinetic parameters of XacGH43_1 obtained by mass spectrometry kinetics. Results are expressed as mean ± SD from three independent experiments.

Substrate	- $CaCl_2$	+ $CaCl_2$
X2	k_{cat}: 16.61±1.05 s^{-1}	k_{cat}: 20.63±1.30 s^{-1}
	K_M: 4.42±0.44 mM	K_M: 2.67±0.19 mM
X6	k_{cat}: 43.41±2.63 s^{-1}	k_{cat}: 25.55±0.67 s^{-1}
	K_M: 3.87±0.37 mM	K_M: 0.40±0.03 mM

Supplementary Table 3: Structural parameters of the characteristic points along the reaction pathway. Distances are given in angstrom and puckering coordinates (θ and ϕ) in degrees. The distances values at each state were computed from all the configurations falling into a small region (±0.1 in terms of the CVs) around the corresponding point in the FEL.

	pathway 1			pathway 2		
	R $^{1}S_{O}$ / $^{2,5}B$	TS $^{2,5}B$	P $^{5}S_{1}$	R $^{4}C_{1}$	TS E_{3}	P $^{4}C_{1}$
C-O1	1.50±0.07	2.40±0.19	3.70±0.05	1.49±0.05	2.74±0.06	3.39±0.06
C1-O_{wat}	3.64±0.07	2.72±0.15	1.48±0.04	3.22±0.08	2.53±0.12	1.48±0.05
O_{wat}-H_{wat}	1.00±0.03	1.01±0.02	1.78±0.05	1.00±0.02	0.98±0.03	1.90±0.07
H_{wat}-O_{D30}	1.80±0.06	1.64±0.16	1.03±0.02	1.79±0.05	1.73±0.09	1.00±0.02
C1-O5	1.39±0.04	1.29±0.02	1.41±0.03	1.41±0.03	1.28±0.01	1.41±0.03
O1-H_{E237}	1.59±0.38	1.03±0.04	1.08±0.16	1.65±0.33	1.03±0.02	1.13±0.15
O_{E237}-H_{E237}	1.15±0.22	1.56±0.04	1.68±0.33	1.10±0.13	1.61±0.09	1.54±0.32
θ	105.81±5.64	99.67±3.12	91.61±2.81	26.64±6.52	34.02±16.34	12.52±6.98
ϕ	142.69±10.88	119.25±4.53	114.32±9.38	157.42±151.68	177.52±13.41	145.18±102.62

Supplementary Table 4: Data collection and refinement statistics.

	XacGH43_1	XacGH43_1 + xylose	XacGH43_1 + xylotriose
Data collection			
PDB code	6XN0	6XN1	6XN2
Space group	$C\,2\,2\,2_1$	$C\,2\,2\,2_1$	$C\,2\,2\,2_1$
Cell dimensions			
a; b; c (Å)	64.56; 165.91; 158.71	65.43; 165.68; 159.11	63.59; 165.87; 158.76
Molecules per AU¶	2	2	2
Resolution (Å)	47.94 – 1.71 (1.77 – 1.71)	48.33 – 1.80 (1.87 – 1.80)	44.61 – 1.65 (1.71 – 1.65)
Total reflections	545,667 (34,204)	385,836 (19,422)	277,556 (6,250)
Unique reflections	90,904 (8,422)	77,797 (6,956)	63,992 (3,113)
$CC_{1/2}$ §	0.99 (0.64)	0.99 (0.75)	0.99 (0.69)
I/σI	14.04 (0.97)	16.74 (1.57)	16.58 (1.04)
Completeness (%)	98.52	97.26	69.60
R_{meas}	0.083	0.062	0.057
Multiplicity	6.0 (4.1)	5.0 (2.8)	4.3 (2.0)
Refinement			
Resolution (Å)	42.00 – 1.71	36.74 – 1.80	29.41 – 1.65
Number of reflections	90,891	77,784	63,968
Number of protein residues	656	662	661
R_{work} / R_{free}	0.171 (0.368) / 0.202 (0.376)	0.173 (0.323) / 0.207 (0.340)	0.177 (0.387) / 0.201 (0.449)
B-factor (Å^2)			
Macromolecules	29.70	25.10	33.90
Ligands	47.20	37.30	47.80
Water	37.30	31.60	37.00
Root mean square deviations			
Bond lengths (Å)	0.014	0.011	0.021
Bond angles (°)	1.46	1.24	1.50
Ramachandran Plot			
Favored (%)	96	96	95
Outliers (%)	0.15	0	0
Molprobity clash score	4.65	3.81	5.22

Statistics for the highest-resolution shell are shown in parentheses.
¶AU: asymmetric unit.
§$CC_{1/2}$: correlation between intensities from random half-datasets[1].

Supplementary References

1. Karplus, P. A. & Diederichs, K. Linking crystallographic model and data quality. *Science (80-.).* **336**, 1030–1033 (2012).
