# Supplementary material for: Two distinct catalytic pathways for GH43 xylanolytic enzymes unveiled by X-ray and QM/MM simulations
Source: Nat Commun. 2021 Jan 14;12:367. doi: 10.1038/s41467-020-20620-3 (PMC7809346; doi:10.1038/s41467-020-20620-3)
Supplement: Supplementary file 3 — Reporting Summary [file 41467_2020_20620_MOESM3_ESM.pdf]

nature research

Corresponding author(s): Mario T. Murakami and Carme Rovira

Last updated by author(s): Dec 7, 2020

Reporting Summary

Nature Research wishes to improve the reproducibility of the work that we publish. This form provides structure for consistency and transparency in reporting. For further information on Nature Research policies, see our Editorial Policies and the Editorial Policy Checklist.

Statistics

For all statistical analyses, confirm that the following items are present in the figure legend, table legend, main text, or Methods section.

n/a	Confirmed	
☐	☒	The exact sample size (*n*) for each experimental group/condition, given as a discrete number and unit of measurement
☐	☒	A statement on whether measurements were taken from distinct samples or whether the same sample was measured repeatedly
☐	☒	The statistical test(s) used AND whether they are one- or two-sided<br>*Only common tests should be described solely by name; describe more complex techniques in the Methods section.*
☒	☐	A description of all covariates tested
☒	☐	A description of any assumptions or corrections, such as tests of normality and adjustment for multiple comparisons
☐	☒	A full description of the statistical parameters including central tendency (e.g. means) or other basic estimates (e.g. regression coefficient) AND variation (e.g. standard deviation) or associated estimates of uncertainty (e.g. confidence intervals)
☐	☒	For null hypothesis testing, the test statistic (e.g. *F*, *t*, *r*) with confidence intervals, effect sizes, degrees of freedom and *P* value noted<br>*Give P values as exact values whenever suitable.*
☒	☐	For Bayesian analysis, information on the choice of priors and Markov chain Monte Carlo settings
☒	☐	For hierarchical and complex designs, identification of the appropriate level for tests and full reporting of outcomes
☒	☐	Estimates of effect sizes (e.g. Cohen's *d*, Pearson's *r*), indicating how they were calculated

Our web collection on statistics for biologists contains articles on many of the points above.

Software and code

Policy information about availability of computer code

Data collection	Crystallographic data were collected using MXCube at MX2-LNLS. CD data were collected with Spectra manager II (Jasco). Dynamic light scattering data were collected using Zetasizer (7.12). MS data were collected using MassLynx (4.1) (Waters). Spectrophotometric data were collected using the i-Control software (1.10.4.0) (Tecan). CZE data were collected using the software 32 Karat 8.0 (Beckman Coulter).
Data analysis	OriginPro (8) was used for non-linear curve fittings. Crystallographic data were indexed and scaled using XDS (version Jun 17, 2015). Molecular replacement was performed using MOLREP from CCP4 package (7.0). Refinements were carried out with phenix.refine (1.8) and REFMAC (5.8), using COOT (0.8.9.1) for manual building. Model validations were done using Molprobity (4.5) and Check My Metal server. Figures containing crystallographic coordinates were generated using Pymol (2.3 or 1.3). SAXS data were integrated using Fit2D (18) and processed using the ATSAS package (2.8) programs. The programs GNOM (5.0), DAMMIN (5.3), DAMAVER (5.0), CRYSOL (2.8.3) and SUPCOMB (2.3) were used for SAXS data processing. Modeller (9) software was used to model missing residues in XacGH43_1 structure. Autodock Vina (1.1.2) and Autodock Tools (1.5.6) were used for molecular docking. Amber18 was used for classical molecular dynamics simulations. Analyses of trajectories were carried out using standard tools of Amber and VMD (1.9.3). QM/MM metadynamics simulations were performed using CPMD (3.15.1) with metadynamics driver provided by the Plumed2 plugin (2.3.3). Free-energy plots were generated using Gnuplot (5).

For manuscripts utilizing custom algorithms or software that are central to the research but not yet described in published literature, software must be made available to editors and reviewers. We strongly encourage code deposition in a community repository (e.g. GitHub). See the Nature Research guidelines for submitting code & software for further information.

Data

Policy information about availability of data

All manuscripts must include a data availability statement. This statement should provide the following information, where applicable:

- Accession codes, unique identifiers, or web links for publicly available datasets
- A list of figures that have associated raw data
- A description of any restrictions on data availability

Structural data have been deposited in the Protein Data Bank (https://www.rcsb.org/) under accession codes 6XN0 (XacGH43_1) [http://doi.org/10.2210/pdb6XN0/pdb], 6XN1 (XacGH43_1 + xylose) [http://doi.org/10.2210/pdb6XN1/pdb] and 6XN2 (XacGH43_1 + xylotriose) [http://doi.org/10.2210/pdb6XN2/pdb]. Source data are provided with this paper. Other data that support this study are available from the corresponding author upon reasonable request.

Field-specific reporting

Please select the one below that is the best fit for your research. If you are not sure, read the appropriate sections before making your selection.

- [x] Life sciences
- [ ] Behavioural & social sciences
- [ ] Ecological, evolutionary & environmental sciences

For a reference copy of the document with all sections, see nature.com/documents/nr-reporting-summary-flat.pdf

Life sciences study design

All studies must disclose on these points even when the disclosure is negative.

Sample size	No sample size calculation was performed in advance. The number of independent experiments (n=3) was determined based upon previous studies with similar methodologies (doi.org/10.1038/s41589-020-0554-5), following the standard practice in enzymology.
Data exclusions	No data were excluded from the analyses.
Replication	All quantitative enzyme assays consist of three independet experiments (n=3). Three independent (n=3) classical molecular dynamics productions were performed for each condition (with or wihtou calcium). Free energy barriers were calculated with two different Gaussian heights for each pre-activated conformation. Calalytic itineraries were also confirmed by isocommittor analysis, where 20 independent unbiased dynamical trajectories with random initial velocities were launched for selected configurations.
Randomization	Randomization was not performed in this work as only in silico molecular dynamics simulations and in vitro biophysical and biochemical experiments were performed.
Blinding	This study reports objective measurements of samples derived from purified proteins, or enzyme reactions. In each experiment, the samples were treated identically. Therefore, blinding was not relevant.

Reporting for specific materials, systems and methods

We require information from authors about some types of materials, experimental systems and methods used in many studies. Here, indicate whether each material, system or method listed is relevant to your study. If you are not sure if a list item applies to your research, read the appropriate section before selecting a response.

Materials & experimental systems

n/a	Involved in the study	
[x]	[ ]	Antibodies
[x]	[ ]	Eukaryotic cell lines
[x]	[ ]	Palaeontology and archaeology
[x]	[ ]	Animals and other organisms
[x]	[ ]	Human research participants
[x]	[ ]	Clinical data
[x]	[ ]	Dual use research of concern

Methods

n/a	Involved in the study	
[x]	[ ]	ChIP-seq
[x]	[ ]	Flow cytometry
[x]	[ ]	MRI-based neuroimaging
